# A Linguistic Analysis of News Coverage of E-Healthcare in China with a Heterogeneous Graphical Model

**DOI:** 10.3390/e24111557

**Published:** 2022-10-29

**Authors:** Mengque Liu, Xinyan Fan, Shuangge Ma

**Affiliations:** 1 School of Journalism and New Media, Xi’an Jiaotong University, No.28 Xianning West Road, Xi’an 710049, China; 2 Center for Applied Statistics, School of Statistics, Renmin University of China, 59 Zhongguancun Street, Haidian District, Beijing 100872, China; 3 Department of Biostatistics, Yale University, 60 College Street, New Haven, CT 06520, USA

**Keywords:** E-healthcare, news coverage, linguistic network, heterogeneous graphical model

## Abstract

E-healthcare has been envisaged as a major component of the infrastructure of modern healthcare, and has been developing rapidly in China. For healthcare, news media can play an important role in raising public interest and utilization of a particular service and complicating (and, perhaps clouding) debate on public health policy issues. We conducted a linguistic analysis of news reports from January 2015 to June 2021 related to E-healthcare in mainland China, using a heterogeneous graphical modeling approach. This approach can simultaneously cluster the datasets and estimate the conditional dependence relationships of keywords. It was found that there were eight phases of media coverage. The focuses and main topics of media coverage were extracted based on the network hub and module detection. The temporal patterns of media reports were found to be mostly consistent with the policy trend. Specifically, in the policy embryonic period (2015–2016), two phases were obtained, industry management was the main topic, and policy and regulation were the focuses of media coverage. In the policy development period (2017–2019), four phases were discovered. All the four main topics, namely industry development, health care, financial market, and industry management, were present. In 2017 Q3–2017 Q4, the major focuses of media coverage included social security, healthcare and reform, and others. In 2018 Q1, industry regulation and finance became the focuses. In the policy outbreak period (2020–), two phases were discovered. Financial market and industry management were the main topics. Medical insurance and healthcare for the elderly became the focuses. This analysis can offer insights into how the media responds to public policy for E-healthcare, which can be valuable for the government, public health practitioners, health care industry investors, and others.

## 1. Introduction

E-healthcare is an emerging field in the intersection of medical informatics, public health, and business and refers to health services and information delivered or enhanced through the Internet and related technologies [1]. As information and communication technologies continue to develop, the E-healthcare industry has been revolutionizing healthcare all over the world. E-healthcare in China began with telemedicine in the 1980s. The Chinese government created the “Internet Plus Healthcare” scheme in 2015 [2] and has issued a series of policies and greenlighted medical institutions to conduct Internet medical services as part of a broader push to promote E-healthcare. The course of policy development can be divided into three periods [3]. The first is the policy embryonic period 2015–2016, during which three policies were issued and marked the promotion of E-healthcare as a national strategy. The second is the policy development period 2017–2019, during which several policies were issued, involving supervision, application, and policy experiment, and focusing on support and guidance from an application perspective. The third is the policy outbreak period 2020–, during which the policies have been mainly focused on the online medical insurance payment standards. With the advancement of technology and promotion of government policies, the E-healthcare industry has been developing rapidly. It has also had an additional boost from the COVID-19 pandemic [4]. We refer to the literature for discussions on technologies adapted to fit local contexts [5], incentives by government organizations [4], legislation and policies [2], fit between E-healthcare systems and work practices or daily clinical work [6], and others.

It is well recognized that news media plays a substantial role in raising public awareness, framing public opinions, and affecting policy formulation and adoption on popular issues [7]. Studies have established the strong associations between media and public’s priorities [8]. For example, McCombs and others [9] showed that media could influence public’s opinions by signaling the importance of certain issues via preferential treatments, such as more frequent coverage and a more prominent position. Guo and McCombs [10] showed that the salience of network relationships among objects and/or attributes, in addition to the ranking of individual elements, could be transferred from media to the public. For healthcare, news media can play an important role in raising public interest and utilization of a particular service [11,12] and complicating (and, perhaps clouding) debate on public health policy issues [13]. For example, Rachul and Caulfield [14] conducted a content analysis of news articles on the access to health therapies and technologies. They found that patients’ perspectives were often highlighted, and that media was largely sympathetic towards patients, thus adding to public debate favoring increased access to healthcare even in the face of equivocal evidence on efficacy.

Published studies have mostly examined the focus and tendency of media coverage on public healthcare issues. “Traditional” qualitative content analysis has been conducted in many studies. For example, Rachul and Caulfield [14] adopted a descriptive coding framework which included the identification of issues discussed in articles and discussion perspectives. Qualitative methods can be time-consuming and inefficient. Some published studies have applied state-of-the-art text mining to analyze news articles and identify coverage and focus [15]. Published quantitative analyses, despite significant successes, often suffered from methodological limitations. For example, Liu and others [16] conducted topic modeling to analyze news articles and identified news coverage and emphases toward public health issues. In their analysis, a document was represented by a bag of words. It neglected the relationships among words and encountered problems such as the loss of semantics and concept ambiguity [17]. Although there were some improvements (for instance, Zolnoori and others [15] considered word order in a text document for topic modeling), the existing studies are still often limited by not sufficiently accommodating the interconnections among words. Furthermore, they have failed to map out the interrelationships among objects and attributes transferred from media to the public, which has high research and practical value [10]. 

In this article, our goal is to conduct a linguistic analysis of news reports on E-healthcare in mainland China. This analysis can advance from the existing literature in multiple important aspects. First, it takes a system perspective and models interconnections. Linguistic networks are analyzed, making this study more powerful than those focusing on individual words and phrases. Network hubs, which represent the focuses of media coverage, are identified. Topics are extracted based on network module detection. The obtained topics can reveal not only debated issues but also interrelationships among objects and attributes transferred from media to the public, which have been missed by the existing works. Second, advancing from some word co-occurrence network studies which focus on words that are consecutive or in the same sentences [18], the HGMND (heterogeneous graphical model for non-negative data) approach adopted in our analysis can accommodate the interconnections among words “far away” from each other. Third, our analysis can effectively model changes over time. The linguistic networks are time-dependent and obtained under a data-driven framework which is more flexible than the previous studies. Under our modeling, change points in time exist and can signal significant events or behaviors (for example, the announcement and implementation of major health policies). With the assistance of penalized estimation, small, spurious changes are “ignored”, and only substantial changes are identified. Between two adjacent change points, networks remain constant. The merit of this study also comes from data analysis, which can offer insight into the temporal patterns of media reports during the process of E-healthcare development in China. Overall, this study can present a novel analysis of news coverage and provide useful information on the media environment of the E-healthcare industry for the government, public health practitioners, and healthcare industry investors. It belongs to the paradigm of information theory analysis, extracts valuable information from mass data, and analyzes using state-of-the-art techniques. It well fits the scheme of this journal.

## 2. Materials and Methods

**Figure 1:**
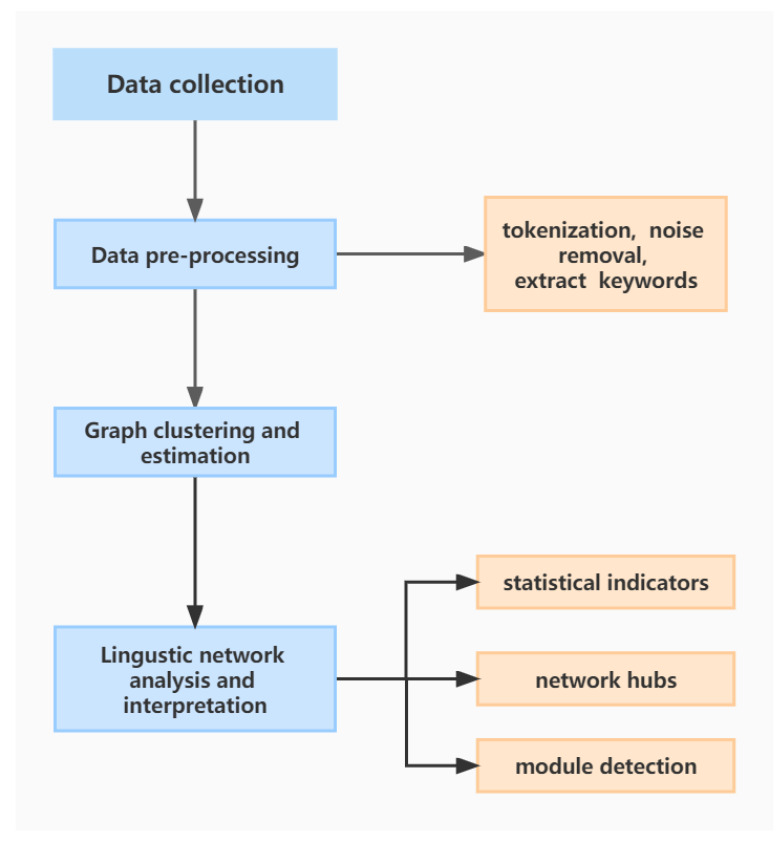
Flowchart of the proposed quantitative linguistic analysis.

The key steps of this research are summarized in Figure 1. 

### 2.1. Data Collection and Pre-Processing

**Figure 2:**
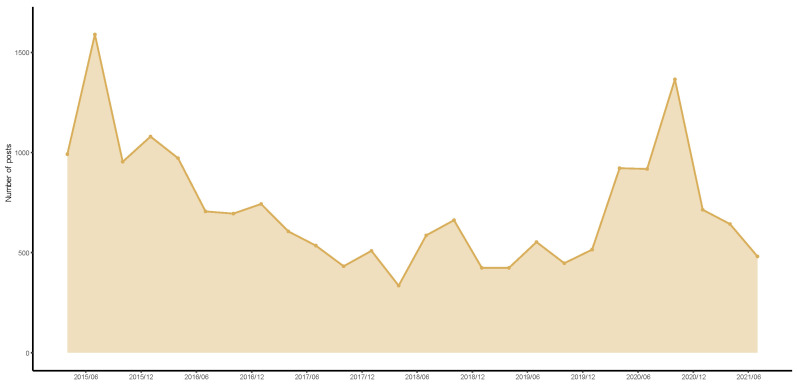
Number of reports related to E-healthcare from January 2015 to June 2021.

Data was retrieved from the WiseOne Intelligent Data Platform (https://www.wisers.com.cn/en/product/intelligentDataPlatform.html, accessed on 1 September 2022). WiseOne is the world’s largest Chinese media information service provider. With a one-stop solution, it covers more than 1500 mainstream media from mainland China, Hong Kong, Macao, Taiwan, Southeast Asia, Europe, and the United States, with a total of more than 30 million news items and over 500,000 articles updated every month. We collected all news reports from newspapers and periodical journals in mainland China related to E-healthcare from January 2015 to June 2021–in China, newspapers and periodical journals are in the category of traditional media. There are a total of 18,804 reports. Figure 2 shows the number of reports for each month, where temporal variations are clearly observed. 

For pre-processing, tokenization was first conducted. Noninformative stopwords were removed. Punctuation marks were excluded. Multi-word tokenization was also conducted to expand a raw token into multiple syntactic words. We extracted the top 100 keywords from the TF-IDF vectors. TF-IDF stands for Term Frequency Inverse Document Frequency of records. It is a weighting system that assigns a weight to each word in a document based on its term frequency (TF) and inverse document frequency (IDF). It is one of the most effective and popular metrics to determine how significant a term is to a text in a series or a corpus. After merging semantically related words, we obtained 51 keywords.

**Figure 3:**
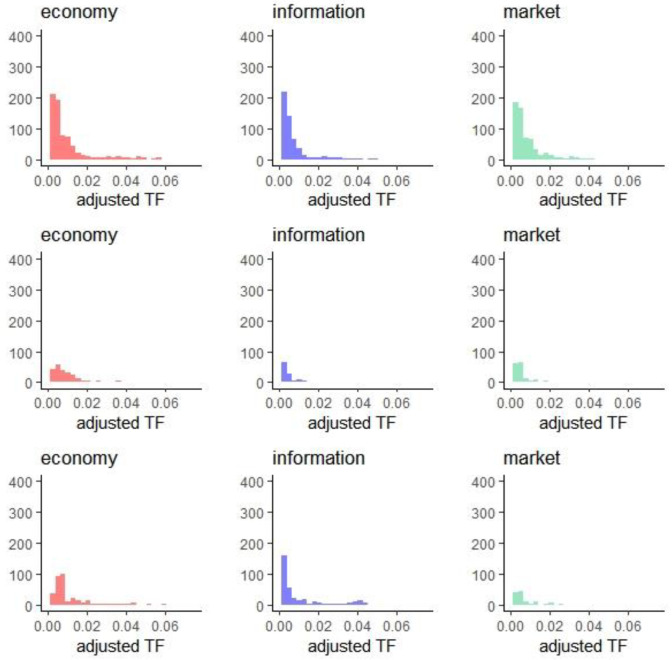
Histograms of adjusted term frequency for selected keywords for 2015 Q1 (**upper**), 2017 Q4 (**center**), and 2019 Q4 (**lower**).

Considering the variation of the number of reports over time, we normalized the term frequencies of the extracted keywords. Specifically, we split the data into multiple subsets, with one subset for each quarter. Then, for each subset/quarter, the adjusted term frequencies of the keywords were considered as the observations of a 51-dimensional random vector. Figure 3 presents the histograms of adjusted term frequency for selected keywords and clearly demonstrates differences in distribution. Differences also exist across the data subsets. 

**Figure 4:**
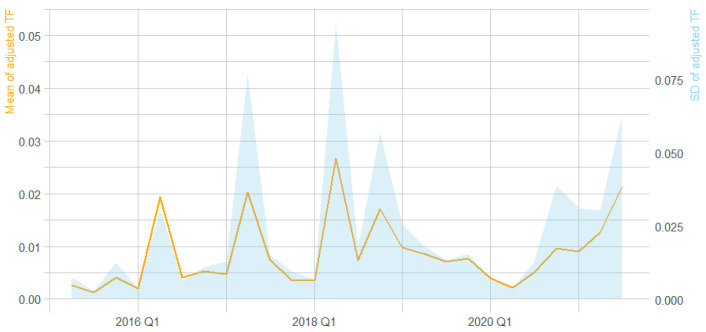
Summary of the adjusted term frequency for the keywords.

Data was further summarized in Figure 4. It was observed that the average adjusted term frequency of the keywords varied over time, and that the standard deviation increased significantly since 2017 Q2. 

### 2.2. Graph Clustering and Estimation

The adjusted term-frequencies of the keywords are non-negative and non-Gaussian, as shown in Figure 2. Observations in each subset can be viewed as homogeneous, while heterogeneity exists across subsets. A non-negative exponential family is adopted to describe data distribution in each subset. Then, the conditional relationships among all keywords in each subset are represented by a parameter matrix. Graphical modeling is a powerful tool for describing conditional dependence relationships among variables. Here, we adopt the HGMND method [19]. In this step, we simultaneously cluster the quarters and estimate a graph for each cluster. 

In the mth subset, the joint density $P_{r}\left(y^{\left(m\right)};\Theta^{*\left(m\right)},\eta^{*\left(m\right)}\right)$ of the adjusted term frequency of keywords vector $Y^{\left(m\right)}={\left(Y_{1}^{\left(m\right)},\ldots ,Y_{p}^{\left(m\right)}\right)}^{T}\in {\mathbb{R}}^{p}$ is assumed to be proportional to:

(1) $$\exp\left\{-\frac{1}{2a}{\left(y^{\left(m\right)}\right)}^{aT}\Theta^{*\left(m\right)}{\left(y^{\left(m\right)}\right)}^{a}+\eta^{*\left(m\right)T}\frac{{\left(y^{\left(m\right)}\right)}^{b}-1_{p}}{b}\right\}I(y^{\left(m\right)}\in {\mathbb{R}}_{+}^{p}),$$

where $y^{\left(m\right)}={\left(y_{1}^{\left(m\right)},\ldots ,y_{p}^{\left(m\right)}\right)}^{T}$ is a realization of $Y^{\left(m\right)}$; matrix $\Theta^{*\left(m\right)}=\left(\theta_{lj}^{*\left(m\right)}\right)\in {\mathbb{R}}^{p\times p}$ and vector $\eta^{*\left(m\right)}=\left(\eta_{j}^{*\left(m\right)}\right)\in {\mathbb{R}}^{p}$ are the true parameters; a,b>0 are known constants; ${\left(y^{\left(m\right)}\right)}^{a}={\left(y_{1}^{\left(m\right)a},\ldots ,y_{p}^{\left(m\right)a}\right)}^{T}$; $1_{p}$ is a p−dimension vector with all elements equal to 1; I(·) is the indicator function; and ${\mathbb{R}}_{+}^{p}={\left[0,\infty \right)}^{p}\;$is the non-negative orthant. In this study, we set a, b=1, and then density (1) corresponds to a truncated Gaussian distribution with $Y^{\left(m\right)}\sim TN\left(\mu^{\left(m\right)},\Sigma^{\left(m\right)}\right)$, where $\Sigma^{\left(m\right)}={\left(\Theta^{*\left(m\right)}\right)}^{-1}$ is positive definite, and $\mu^{\left(m\right)}$=$\Sigma^{\left(m\right)}\eta^{*\left(m\right)}$. 

For estimation, we consider the objective function:

(2) $$\hat{L}\left(\left\{\Theta \right\},\left\{\eta \right\}\right)=\sum_{m=1}^{M}\hat{J}\left(\Theta^{\left(m\right)},\eta^{\left(m\right)}\right)+P(\left\{\Theta \right\};E),$$

where $\left\{\Theta \right\}=\left\{\Theta^{\left(m\right)\;},\;m=1,\;.\;.\;.\;,\;M\right\}$ and $\left\{\eta \right\}=\left\{\eta^{\left(m\right)}\;,\;m=1,\;.\;.\;.\;,\;M\right\}$ are the parameters of the joint density in the form of (1) to approximate the true density. The first term of (2) is the sum of M loss functions, where $\hat{J}\left(\Theta^{\left(m\right)},\;\eta^{\left(m\right)}\right)$ is the empirical generalized h-score matching loss for the mth dataset. For more details on $\hat{J}\left(\Theta^{\left(m\right)},\;\eta^{\left(m\right)}\right)$, we refer to [19]. The second term P({Θ}; E) is a penalty function, and we adopt:

$$P\left(\left\{\Theta \right\};E\right)=\lambda_{1}\sum_{m=1}^{M}\left|\Theta^{\left(m\right)-}\right|+\lambda_{2}\sum_{m=1}^{M}{\left\|\Theta^{\left(m\right)-}-\Theta^{\left(m+1\right)-}\right\|}_{F},$$

where $\Theta^{\left(m\right)-}=\Theta^{\left(m\right)}-diag\left(\theta_{11}^{\left(m\right)},\ldots ,\theta_{pp}^{\left(m\right)}\right)$, $\left|\Theta^{\left(m\right)-}\right|=\underset{l\neq j}{\sum }\left|\theta_{lj}^{\left(m\right)}\right|$, ${\left\|\cdot \right\|}_{F}\;$is the Frobenius norm, and $\lambda_{1},\lambda_{2}$ are the non-negative tuning parameters. This penalty function has two terms. The first term can lead to sparsity for $\Theta^{\left(m\right)},\;m=1,\ldots ,M$. The fused group Lasso term penalizes the difference in conditional relationships between two datasets in adjacent quarters. It can borrow strength across the datasets in adjacent quarters when estimating $\Theta^{\left(m\right)}$. Furthermore, it can help identify the cluster structure (change points) of $\Theta^{\left(m\right)}$’s. Specifically, if, for example, $\Theta^{\left(m_{1}\right)-}\neq \Theta^{\left(m_{1}+1\right)-}=\cdots =\Theta^{\left(m_{1}+k\right)-}\neq \Theta^{\left(m_{1}+k+1\right)-}$, we conclude that the conditional relationships of the keywords in the $\left(m_{1}+1\right)$th time unit to the $\left(m_{1}+k\right)$th time unit are the same, and that the $\left(m_{1}+1\right)$th to $\left(m_{1}+k\right)$th time units belong to the same cluster. Besides, $m_{1}$ and $m_{1}+k$ are two change points. Tuning parameter $\lambda_{2}$ controls the degree of heterogeneity among the estimates of $\Theta^{\left(m\right)}$’s. For tuning parameter selection, we adopt an AIC-type criterion [19] and a grid search.

In principle, it is possible that non-adjacent time units/datasets have the same $\Theta^{\left(m\right)-}$ values, although we note that such a model structure may be hard to interpret. When it is desirable to allow for such a structure, we can replace the second term of the penalty function with a pairwise penalty $\lambda_{2}\underset{m_{1}<m_{2}}{\sum }{\left\|\Theta^{\left(m_{1}\right)-}-\Theta^{\left(m_{2}\right)-}\right\|}_{F}$. For more details, we refer to [20]. 

### 2.3. Linguistic Network Analysis and Interpretation

Based on the graphs obtained above, linguistic networks can be constructed. The linguistic network for the mth graph can be expressed as $G^{\left(m\right)}=\left(V^{\left(m\right)},\;E^{\left(m\right)}\right)$, where $V^{\left(m\right)}$ is a set of nodes (where each node represents a keyword), and $E^{\left(m\right)}$ is a set of edges. Edge $e_{ij}^{\left(m\right)}\in E^{\left(m\right)}$ connects nodes i and j if ${\hat{\theta }}_{ij}^{\left(m\right)}\neq 0,\;$which means that the two keywords have a conditional dependence relationship. The number of edges is denoted as $q^{\left(m\right)}=\left|E^{\left(m\right)}\right|$, and $n^{\left(m\right)}=n=\left|V^{\left(m\right)}\right|$ denotes the number of nodes. We examine statistical properties and structures of the linguistic networks from three aspects: statistical indicators, network hubs, and modules.

(1) Statistical indicators

We examine a set of informative statistical indicators associated with the topological properties of the linguistic networks:

1. The average degree for the mth network is computed as:
  $$<k_{i}^{\left(m\right)}>=\frac{1}{n}\sum_{i=1}^{n}k_{i}^{\left(m\right)},$$
  where <> is sample average, $k_{i}^{\left(m\right)}$ is the degree of node *i,* and $k_{i}^{\left(m\right)}=\left|\left\{j\;\in V^{\left(m\right)}|\;\left\{i,\;j\right\}\in \;E^{\left(m\right)}\right\}\right|$.
2. The average shortest-path length (*ASPL*) is the average value of the shortest-path length between any two nodes in the network. For the mth network, it is calculated as:
  $$ASPL^{\left(m\right)}=\frac{2\sum_{i>j}d_{ij}^{\left(m\right)}}{n\left(n-1\right)},$$
  where $d_{ij}^{\left(m\right)}\;$is the shortest-path length between nodes *i* and *j*.
3. The clustering coefficient is the average of the clustering coefficients of all nodes. For the mth network, it is defined as:
  $$CC^{\left(m\right)}=\frac{1}{n}\sum_{i}\frac{q_{i}^{\left(m\right)}}{k_{i}^{\left(m\right)}\left(k_{i}^{\left(m\right)}-1\right)/2},$$
  where $k_{i}^{\left(m\right)}$ is the degree of node i, and $q_{i}^{\left(m\right)}$ is the number of edges among the $k_{i}^{\left(m\right)}$ neighbor nodes. For an Erdös–Renyi network, the average shortest-path length is $ASPL_{r}^{\left(m\right)}\approx \;\ln\left(n\right)/\left(\ln\left(2q^{\left(m\right)}\right)-\ln\left(n\right)\right)$, and the clustering coefficient is $CC_{r}^{\left(m\right)}\approx \;2q^{\left(m\right)}/n\left(n-1\right)$. A network is said to have the small-world property if the small-world coefficient σ=($CC^{\left(m\right)}/CC_{r}^{\left(m\right)})/$($ASPL^{\left(m\right)}/ASPL_{r}^{\left(m\right)})$ > 1 [21].
4. The degree distribution, for the mth network, is denoted as $P\left(k^{\left(m\right)}\right)\;$and is the probability that a randomly chosen node has exactly degree $k^{\left(m\right)}.\;$In case $P\left(k^{\left(m\right)}\right)$ satisfies the power-law degree distribution, that is, $P\left(k^{\left(m\right)}\right)\;\propto k^{\left(m\right)}{}^{-\gamma }$ where γ is a positive constant, the network is called scale-free [22].

(2) Network hubs

Degree centrality and eigenvector centrality are used to identify network hubs. This allows us to evaluate the qualitative nature of the most central words in the networks. For our analysis, network hubs can reflect the focuses of media coverage which may lead the public to consider those issues as important. 

For a node, the degree centrality is its degree, and the eigenvector centrality is based on the centrality of its neighbors. For the mth network, the eigenvector centrality for node *i* is the *i*th element of $x^{\left(m\right)}$, defined by:

$$A^{\left(m\right)}x^{\left(m\right)}=\lambda^{\left(m\right)}x^{\left(m\right)},$$

where $A^{\left(m\right)}$ is the adjacency matrix of graph $G^{\left(m\right)}\;$with eigenvalue $\lambda^{\left(m\right)}$, $A_{ij}^{\left(m\right)}$ = 1 if node *i* is linked to node *j*, and $A_{ij}^{\left(m\right)}$ = 0 otherwise. Based on the Perron–Frobenius theorem, there is a unique solution $x^{\left(m\right)}$, all of whose entries are positive, if $\lambda^{\left(m\right)}$ is the largest eigenvalue of the adjacency matrix [23].

(3) Module detection

A module is defined as a set of densely connected nodes that are sparsely connected to the other modules in the network. The Louvain algorithm [24] is adopted and realized using the Gephi software (version 0.9.7). A modularity score is maximized by choosing an appropriate division of the network. More specifically, for the mth network, the modularity score is computed as:

$$Q^{\left(m\right)}\left(c\right)=\frac{1}{2l^{\left(m\right)}}\underset{ij}{\sum }\left[A_{ij}^{\left(m\right)}-\lambda \frac{k_{i}^{\left(m\right)}k_{j}^{\left(m\right)}}{2l^{\left(m\right)}}\right]\delta_{ij}^{\left(m\right)}\left(c\right),$$

where c is a partition of the nodes, $l^{\left(m\right)}$ is the total number of edges, $k_{i}^{\left(m\right)}$ is the degree of node *i*, and λ is a tuning parameter. $\delta_{ij}^{\left(m\right)}\left(c\right)$ describes whether nodes *i* and *j* are in the same module: if yes, $\delta_{ij}^{\left(m\right)}\left(c\right)=1$; otherwise, $\delta_{ij}^{\left(m\right)}\left(c\right)$ = 0. 

**Figure 5:**
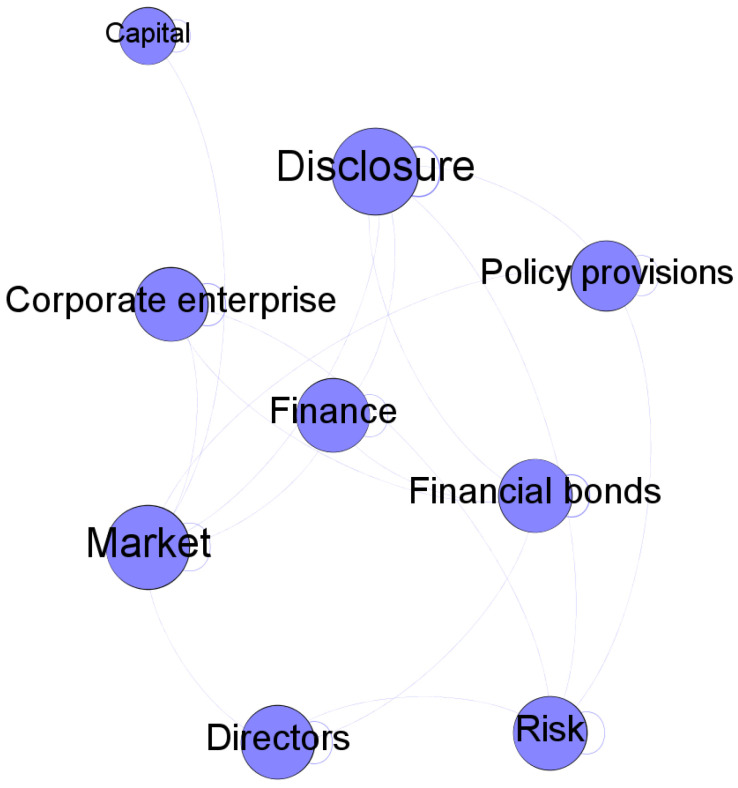
A sample module.

The Louvain algorithm can unfold a complete hierarchical modular structure of a network, thereby allowing for different resolutions of module detection. In Gephi, the resolution parameter, which describes how much between-group edges impact the modularity score, determines the granularity level at which modules are detected [25], with a low-resolution value resulting in more modules. The analysis of associations between words in a module can assist keeping track of semantic structures. The words in the same module are likely to describe tightly connected topics. We extract topics based on semantic structures of the detected modules. For example, most of the words in Figure 5 are related to financial market. As such, this module can be considered as describing financial market. The detected topics can reveal not only debated issues but also interrelationships among objects and attributes transferred from media to the public. 

To describe the strengths of topics, we consider two measures: the absolute and relative strengths. Assume that U topics are extracted based on module detection. Following [17,26], for the mth quarter, the absolute and relative strengths of topic *u* can be computed as:

$$\mathrm{AStrength}\left(u,m\right)=\frac{n_{u,m}\;}{M_{m}},\;\mathrm{RStrength}\left(u,m\right)=\frac{\frac{n_{u,m}\;}{M_{m}}}{\sum_{u=1}^{U}\frac{n_{u,m}\;}{M_{m}}},$$

where $M_{m}$ and $n_{u,m}$ are the total number of news and the number of news related to topic u in the mth quarter, respectively.

## 3. Results

### 3.1. Graph Clustering and Estimation

**Figure 6:**
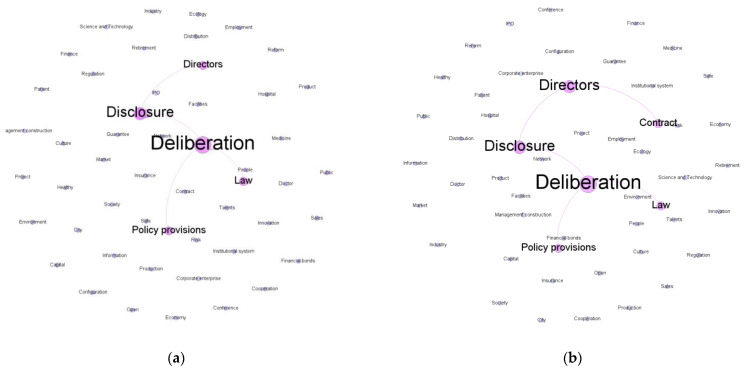

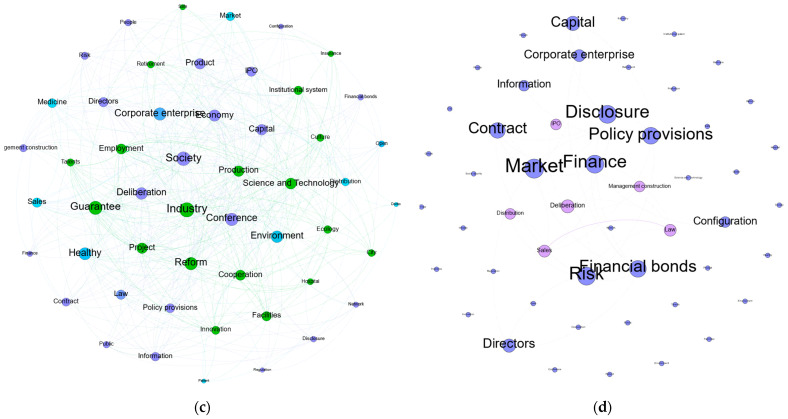
Conditional dependence relationships among the keywords (2015 Q1–2019 Q2). (**a**) 2015 Q1–2015 Q4. (**b**) 2016 Q1–2017 Q2. (**c**) 2017 Q3–2017 Q4. (**d**) 2018 Q1–2019 Q2.

**Figure 7:**
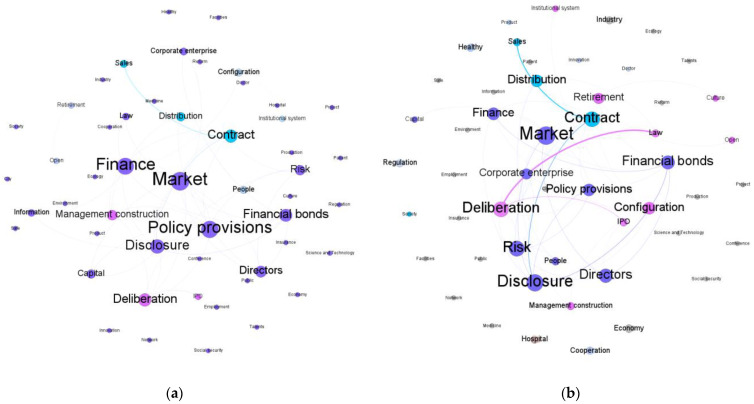

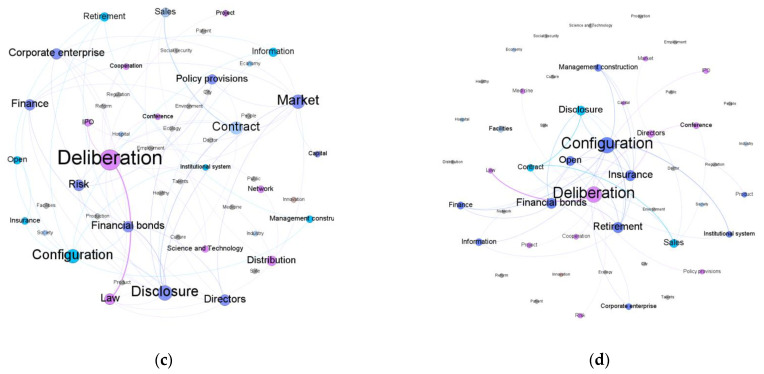
Conditional dependence relationships among the keywords (2019 Q3–2021 Q2). (**a**) 2019 Q3. (**b**) 2019 Q4–2020 Q1. (**c**) 2020 Q2. (**d**) 2020 Q3–2021 Q2.

Figure 6 and Figure 7 present the clustering of quarters and the graphs illustrating the conditional dependence relationships among the keywords. 

Based on the graph clustering and estimation results, we found that there were eight phases of media coverage. Specifically, in the policy embryonic period 2015–2016, two phases were observed, their estimated graphs are sparse, and a limited number of issues were discussed. The policy development period 2017–2019 was divided into four phases. Among them, the estimated graph for the 2017 Q3–2017 Q4 phase is the densest. The 2019 Q4–2020 Q1 phase can be viewed as the transition phase between the policy development and outbreak periods. Two phases were discovered in the policy outbreak period 2020–.

### 3.2. Linguistic Network Analysis

#### 3.2.1. Statistical Indicators

Summary information is provided in Table 1. Based on this, an overview of the linguistic networks can be obtained. The average degrees of the networks in 2015 Q1–2015 Q4 and 2016 Q1–2017 Q2 are much lower, which suggests much sparser networks. It has the highest value in 2017 Q3–Q4, and those in the other phases are similar. 

The small-world coefficient σ, calculated based on the average shortest-path length and clustering coefficient, was larger than 1 in the phases after 2018 Q1. This suggests the presence of the small-world phenomenon in the linguistic networks of those phases. 

In the analysis of degree distribution, it was found that all networks, except for that in 2017 Q3–2017 Q4, exhibited power-law degree distributions, with the power-law exponent ranging between 2 and 2.6. This scale-free characteristic suggested that the connectivity values of a small number of nodes were large (with many connections), rendering them leading roles in the networks. On the other hand, most other nodes had limited connections. The degrees of the network in 2017 Q3–2017 Q4 followed a skewed bell-shaped distribution.

#### 3.2.2. Network Hubs 

For each linguistic network, the five most central hubs are listed in Table 2. It is noted that the degree and eigenvector centrality captured quite similar patterns. Among the hubs, words such as “Deliberation”, “Directors”, “Disclosure” reflect the focus on regulation. They were at the center of the networks in most phases. “Law” and “Policy provisions”, reflecting the focus on policy, were the central network hubs in the policy embryonic period 2015–2016. Words related to finance and economy, such as “Finance”, “Financial bonds”, “Risk”, “Market”, “Contract”, became the most central network hubs in 2018 Q1, which was the middle of the policy development period. In the policy outbreak period 2020–, “Insurance”, “Retirement”, which were related to specific healthcare domains, were the most central words. 2017 Q3–2017 Q4 was the opening phase of the policy development period. The hubs in that phase were quite different from those in the other phases and included “Industry”, “Society”, “Reform”, “Healthy”, “Conference”, “Guarantee”, which were related to a wide range of issues. 

Overall, in the policy embryonic period 2015–2016, the media focused on policy and regulation. In the policy development period 2017–2019, the major focuses of media coverage included social security, healthcare and reform, etc. in 2017 Q3–2017 Q4. Since 2018 Q1, industry regulation and finance had become the focuses. In the policy outbreak period 2020–, medical insurance and healthcare for the elderly started to be the focal points.

#### 3.2.3. Module Detection

**Figure 8:**
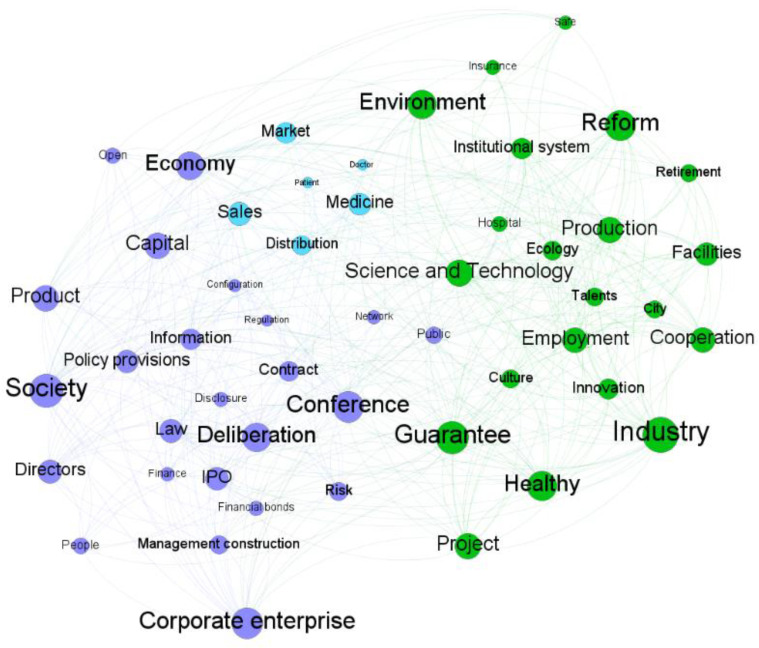
Module detection for the linguistic network of 2017 Q3–2017 Q4.

**Figure A1:**
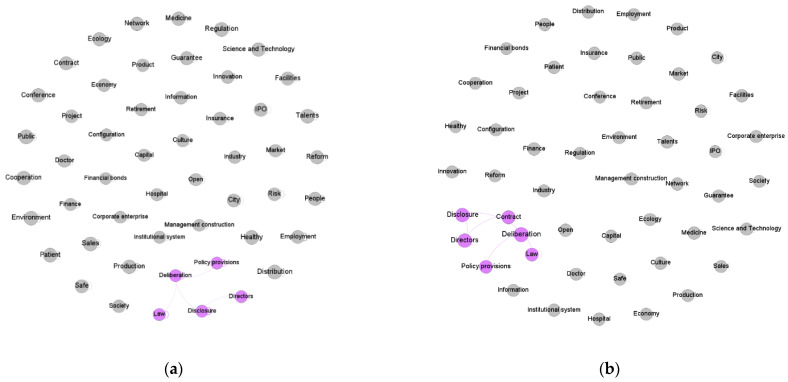

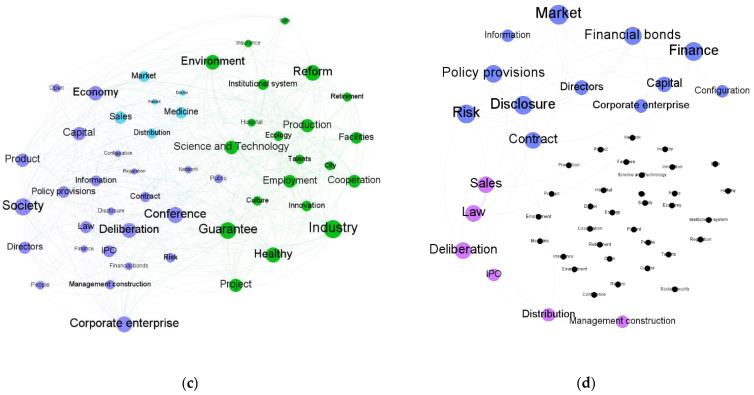
Module detection for the linguistic networks (2015 Q1–2019 Q2). (**a**) 2015 Q1–2015 Q4. (**b**) 2016 Q1–2017 Q2. (**c**) 2017 Q3–2017 Q4. (**d**) 2018 Q1–2019 Q2.

**Figure A2:**
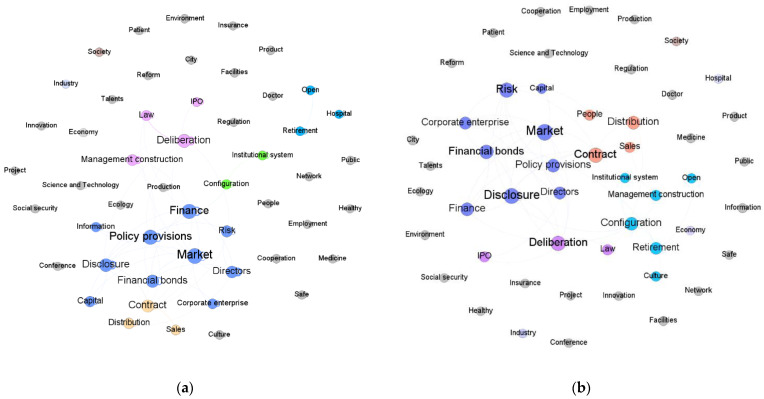

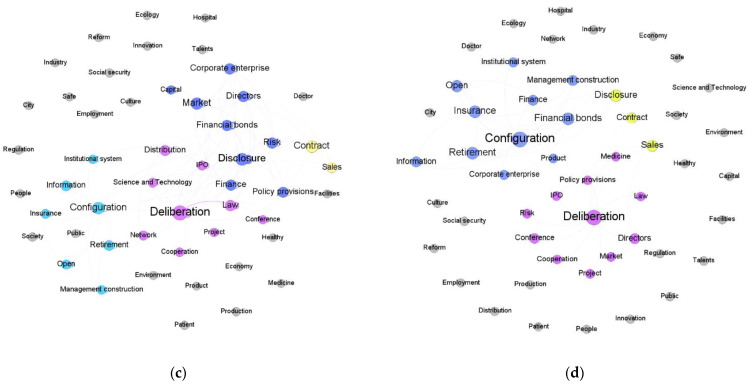
Module detection for the linguistic networks (2019 Q3–2021 Q2). (**a**) 2019 Q3. (**b**) 2019 Q4–2020 Q1. (**c**) 2020 Q2. (**d**) 2020 Q3–2021 Q2.

Take the linguistic network for 2017 Q3–2017 Q4 as an example. Under the default resolution value of 1.0, there were 3 modules as presented in Figure 8, where different modules were represented with different colors, and the modularity value was 0.337. As shown in Table 3, we extracted three topics from the detected modules. For the modules, the average clustering coefficient was 0.455, suggesting a significant clustering effect. The same analysis was conducted on the other phases. The module detection results and the summary of module detection are provided in Appendix A Figure A1 and Figure A2 and Table A1, respectively. Modules with fewer than three words were omitted to improve presentation. Note that, the modularity values were smaller than 0.3 for 2015 Q1–2015 Q4 and 2016 Q1–2017 Q2, suggesting less satisfactory partitioning of the networks. The two corresponding linguistic networks were sparse, and as such, we extracted the topics directly from the semantic structures.

**Figure A3:**
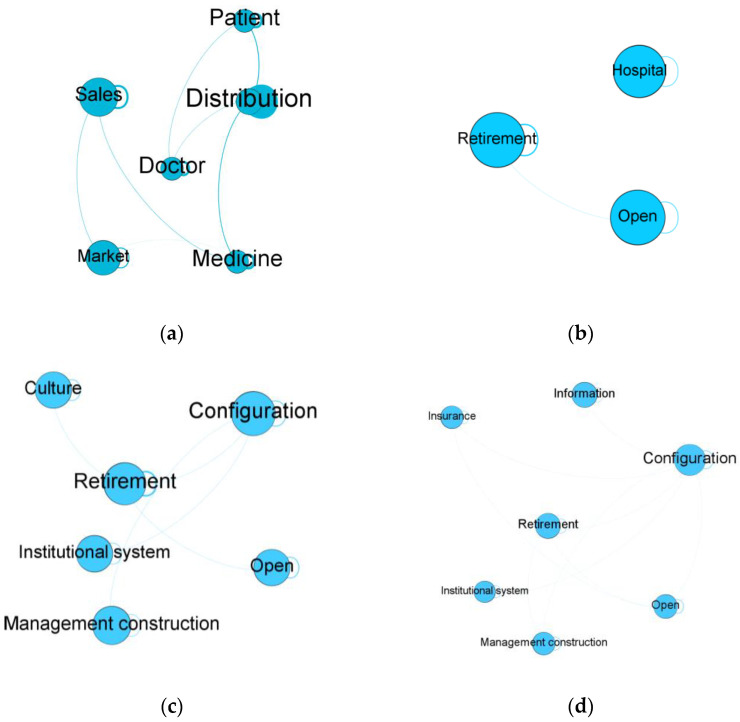
Semantic structure of Topic 2. (**a**) 2017 Q3–2017 Q4. (**b**) 2019 Q3. (**c**) 2019 Q4–2020 Q1. (**d**) 2020 Q2.

**Figure A4:**
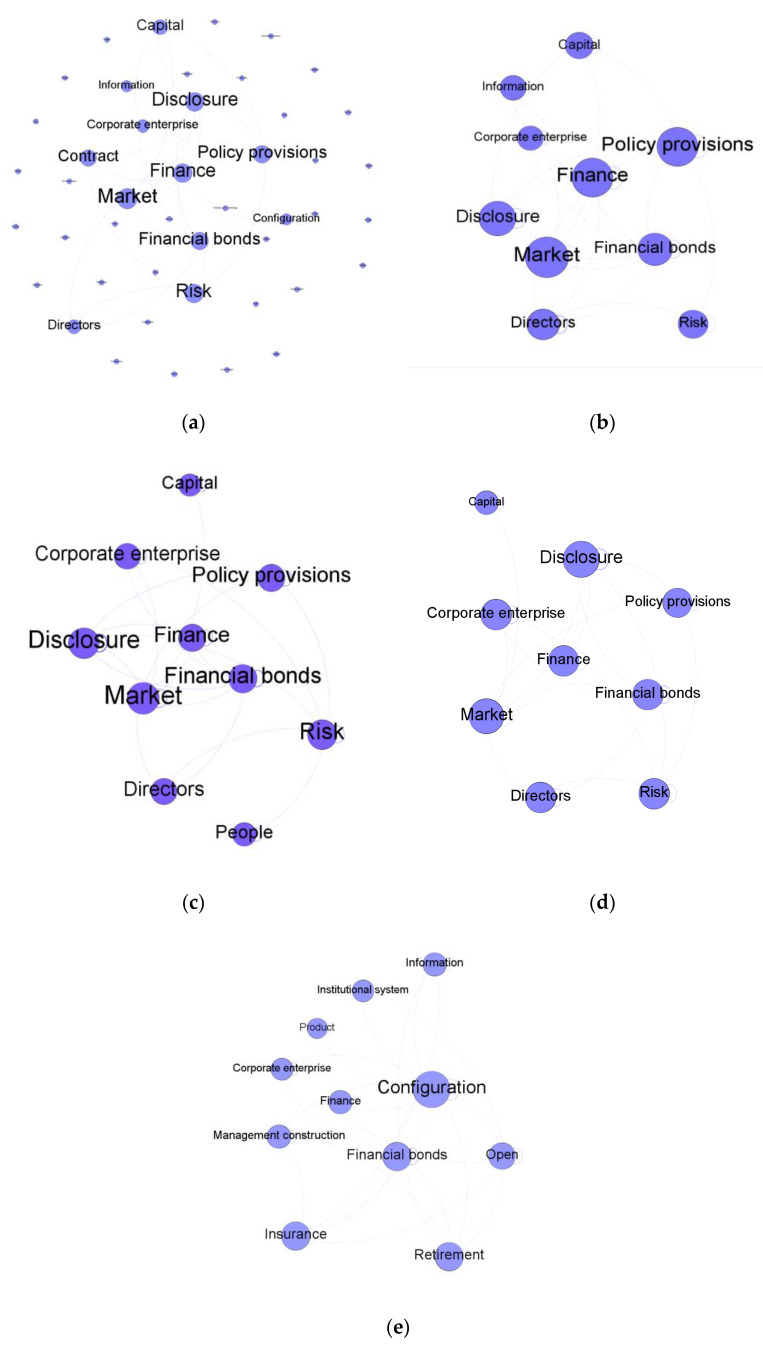
Semantic structure of Topic 3. (**a**) 2018 Q1–2019 Q2. (**b**) 2019 Q3. (**c**) 2019 Q4–2020 Q1. (**d**) 2020 Q2. (**e**) 2020 Q3–2021 Q2.

**Figure A5:**
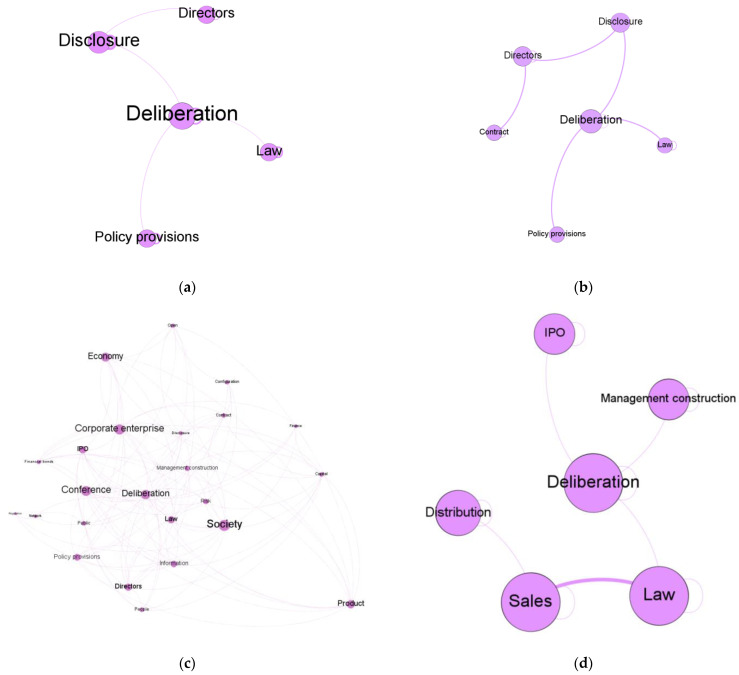
Semantic structure of Topic 4 (2015 Q1–2019 Q2). (**a**) 2015 Q1–2015 Q4. (**b**) 2016 Q1–2017 Q2. (**c**) 2017 Q3–2017 Q4. (**d**) 2018 Q1–2019 Q2.

**Figure A6:**
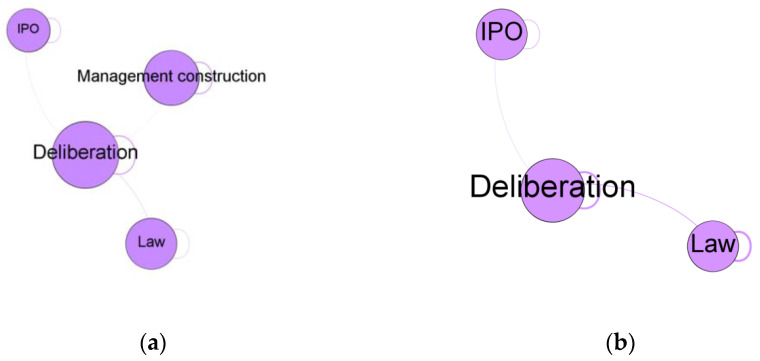

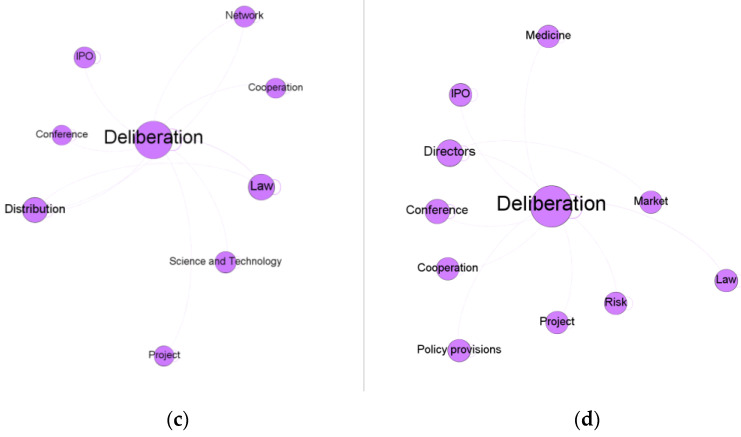
Semantic structure of Topic 4 (2019 Q3–2021 Q2). (**a**) 2019 Q3. (**b**) 2019 Q4–2020 Q1. (**c**) 2020 Q2. (**d**) 2020 Q3–2021 Q2.

Overall, four main topics were summarized based on the detected modules, namely, Topic 1: industry development, Topic 2: health care, Topic 3: financial market, and Topic 4: industry management. The topic structures of different phases are presented in Table 4. The detailed semantic structures are presented in Appendix A Figure A3, Figure A4, Figure A5 and Figure A6.

In the policy embryonic period 2015–2016, the media coverage had two phases, and Topic 4: industry management was the main topic. As shown in Appendix A Figure A5, the interrelationships among the words transferred from media to the public were similar in these two phases. The most central hub “Deliberation” was interconnected with “Law”, “Policy provisions”, and “Disclosure”. 

**Figure 9:**
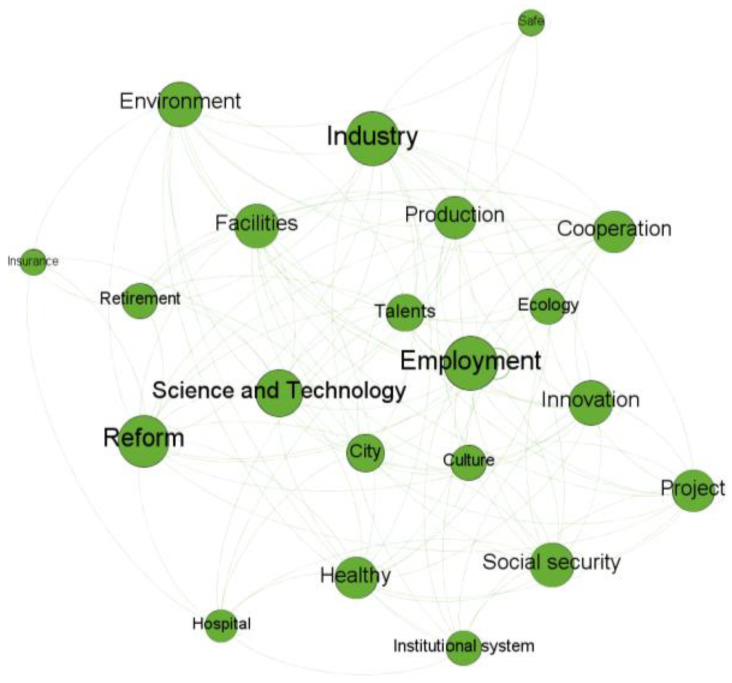
Topic 1 in 2017 Q3–Q4.

In the policy development period 2017–2019, all the four main topics were detected. Among them, Topic 1: industry development became a debated issue only in 2017 Q3–2017 Q4. As shown in Figure 9, “Industry” was interconnected with “Employment”, “Retirement”, “Talents”, “Reform”, “Facilities”, “production”, “Science and technology”, etc. As shown in Figure A3, in 2017 Q3–Q4, Topic 2 was related to medical sales. “Patient”, “Medicine”, “Distribution”, “Doctor”, “Sales”, and “Market” were interconnected with each other. In 2019 Q3, “Retirement” was interconnected with “Hospital” and “Open”. In 2019 Q4–2020 Q1, “Retirement”, “Culture”, “Configuration”, and “Institutional system” were interconnected with each other. Since 2019 Q3, Topic 2 was mainly about healthcare for the elderly. Topic 3: financial market became a key issue in 2018 Q1. “Finance”, “Financial bonds”, “Risk”, “Market”, ”Policy provisions”, and “Disclosure” were interconnected with each other. Topic 4: industry management was also a key point of discussion. However, the interrelationships among the words changed in the policy development period. “Economy”, “Deliberation”, “Conference”, “Society”, “Risk”, “IPO”, “Finance”, “Capital”, “Law” and “Corporate enterprise” were interconnected with each other in 2017 Q3–2017 Q4. Topic 4 in that phase was related to a wide range of issues. The number of words included in Topic 4 sharply reduced in the remaining phases of that period. For example, only “IPO”, “Deliberation”, “Law”, and “Management construction” were included in 2019 Q3.

In the policy outbreak period 2020–, the policies were mainly focused on the payment standards of online medical insurance and comprehensively promoted the implementation of online medical treatment. In 2020 Q2, as presented in Appendix A Figure A3, “Insurance” was included in Topic 2. As shown in Appendix A Figure A4, Figure A5 and Figure A6, in 2020 Q3–2021 Q2, “Insurance” and “Retirement” were included in Topic 3, which was mainly about health insurance and financial risk in that phase. In 2020 Q3–2021 Q2, “Medicine” was included in Topic 4. Medicine management became a concern in 2020 Q3–2021 Q2. 

**Figure 10:**
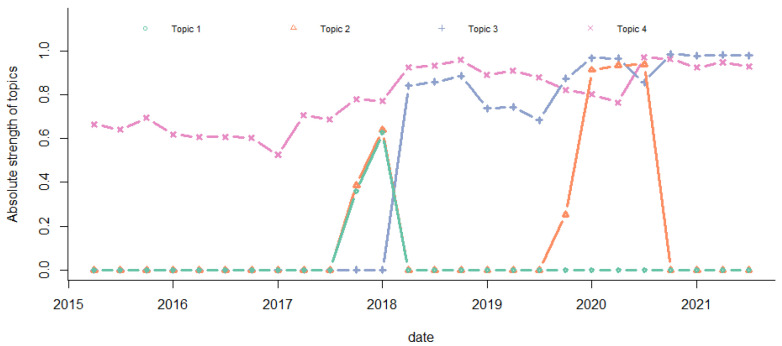
Absolute strength of topics.

**Figure 11:**
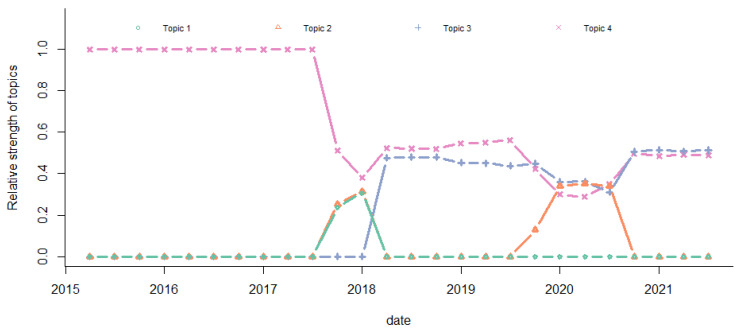
Relative strength of topics.

The absolute and relative strengths of the four main topics over time are plotted in Figure 10 and Figure 11, respectively. We observed that the absolute strength of Topic 4 increased over time. It had the highest level before 2019 Q3. Topic 3 surpassed Topic 4 from 2019 Q3. The absolute strength of Topic 1 was the lowest. In 2019 Q4–2020 Q2, the strength levels of Topic 2, Topic 3, and Topic 4 were similar. The relative strengths had the same pattern.

## 4. Discussion

E-healthcare has played and may play an even more important role in modern healthcare. The open nature of internet requires that organizational policies and procedures be put in place to ensure the privacy, integrity, and quality of E-healthcare systems. In China, the E-healthcare industry is supervised and managed by various regulatory agencies. Mass media is an important venue connecting the policy system and the public. Our data analysis can offer insight into the temporal patterns of media reports during the process of E-healthcare development in China. 

The linguistic network analysis results can help understand how the media responds to public policy during the process of E-healthcare policy issuance and implementation. Some published studies suggested that the traditional media might not be significantly affected by some policy announcements and partial implementations in China [27,28]. For E-healthcare and some other domains, the impact of the public policy process on traditional media has not yet been well analyzed [27]. Our results suggest that the temporal patterns of media reports have been mostly consistent with the policy trend. Specifically, in the policy embryonic period 2015–2016, on 15 October 2016, the State Council issued the outline of “Healthy China 2030”, which was the first time that E-healthcare was mentioned at the national strategy level. Industry management was the main topic of debate. In the policy development period 2017–2019, several policies and government plans on multiple levels of supervision and technology application were successively issued, focusing on support and guidance at the application level. E-healthcare policies entered a period of development. Many more topics needed to be thoroughly discussed with a series of related policies published. All the four main topics, namely industry development, healthcare, financial market, and industry management, were present in that period. This reflects the characteristics and functionalities of media. 2017 Q3–2017 Q4 was the opening phase of the policy development period and also the phase with the most extensive media debate. The issues on E-healthcare industry development were discussed only in that phase and were closely linked to employment, retirement, organizational reform, and scientific and technological development. The debate on industrial management covered many areas, such as corporate management, law, and finance. Since 2018 Q1, the development path of the E-healthcare industry had gradually become clear. In April 2018, the General Office of the State Council issued “Opinions on Promoting the Development of “Internet Plus Healthcare”, “which was a programmatic policy of E-healthcare. Supporting policies of Internet diagnosis and treatment, Internet hospital and telemedicine were successively published to further clarify the issue of Internet medical charges [2]. Meanwhile, since 2018 Q1, the debate and concerns on finance and industry regulation had become the focuses of media coverage. In April 2019, the State Council published “Opinions on Promoting the Development of Elderly Care Services”. Specific policies were put forward to implement the “Internet Plus elderly care” initiative and improve the healthcare system for the elderly. Since 2019 Q3, healthcare for the elderly had begun to attract media and the public’s attention, which was associated with the huge market demand of E-healthcare for the elderly in China. Online payment for medical insurance was a challenge that restricted the development of E-healthcare. In August 2019, the National Healthcare Administration issued “Guidance on Improving the “Internet Plus” Medical Service Price and Medical Insurance Payment”, which included E-healthcare services into medical insurance coverage for the first time. In February 2020, online payment for medical insurance was first experimented in the Zhejiang province, marking a significant breakthrough. With the announcement and implementation of the medical insurance payment policy, the “medical + medicine + medical insurance” linkage mechanism was promoted. In the policy outbreak period 2020-, the media continued to align with the policies, which focused on the payment standards of online medical insurance and financial management. Medicine management started to become a concern in 2020 Q3. 

During the study period, the COVID-19 pandemic had a strong impact on E-healthcare and the whole healthcare industry. The absolute strength of Topic 2: health care peaked in 2020 Q1 and 2020 Q2, and the absolute strength of Topic 4: industry management increased significantly in 2020 Q2, both of which might be associated with the pandemic. However, the worst period of the COVID-19 pandemic in China was relatively short, and thus its impact on the focuses and main topics of E-healthcare media coverage was not that strong. 

In China, more detailed policies and actions that can promote the development of crucial links of E-healthcare, such as prescription circulation, medical insurance payment and online medical sales, are still needed. It is imperative that research institutes, social organizations, public health practitioners, and health care industry investors can get involved in the debate on E-healthcare issues and related policies. Our findings can provide insight into the media environment of E-healthcare industry for them. 

### Limitations

This study inevitably has limitations. Our data source was limited to traditional media. According to Cui [29], there are currently three main categories of media in China: traditional media, internet media, and mobile media. Traditional media is relatively more regulated, while internet media and mobile media can be more convenient for public debate. Most of our extracted keywords are nouns, and only a few are adjectives. These two types of words may play different roles. For example, unlike nouns, the adjective words were observed to be rarely interconnected with each other. The adjectives were interconnected with the nouns and described the nature and direction of discussion on related issues. Additionally, compared to the nouns, the adjectives had fewer links and were farther from the center of the network in most of the phases. It has been suggested that adjectives may differ from nouns in linguistic networks. With a small number of adjectives, we have not separately considered nouns and adjectives. Our analysis has been focused on media, and the observed changes have been explained from a policy change perspective. It has been recognized that media can also impact policy–as such, media and policy can be viewed as one interacting system. We defer to future research to study this system and conduct quantitative (as opposed to qualitative as in this study) analysis of the impact of policy change. Additionally, network-based analysis is still evolving, and the adopted analysis techniques may not be “optimal” for the proposed analysis.

## 5. Conclusions

A linguistic analysis of news reports on E-healthcare has been conducted. With the high significance of E-healthcare in China and worldwide and limited published linguistic analysis, this study is warranted. The data collection has broadly covered a large number of reports and from January 2015 to June 2021. A state-of-the-art network analysis technique has been adopted. The novel modeling has led to the discovery of highly informative data characteristics not achieved in the existing analyses. As discussed above, the findings are highly sensible and have demonstrated the alignment of media coverage with policy development and changes. Despite minor limitations, this study can be highly informative for multiple stakeholders.

## Figures and Tables

**Table 1:** Summary of the keyword networks.

Phase	Average Degree	$ASPL/ASPL_{r}$	$CC/CC_{r}$	σ	γ
2015 Q1–2015 Q4	0.157	1.800/3.424	0.000/0.400	0.000	2.328
2016 Q1–2017 Q2	0.196	2.133/3.507	0.000/0.333	0.000	2.077
2017 Q3–2017 Q4	18.275	1.711/1.353	0.455/0.365	0.986	---
2018 Q1–2019 Q2	1.529	2.118/1.971	0.303/0.254	1.110	2.496
2019 Q3	1.725	2.099/2.229	0.385/0.190	2.151	2.285
2019 Q4–2020 Q1	2.039	2.411/2.539	0.302/0.137	2.321	2.213
2020 Q2	2.510	2.268/2.118	0.559/0.182	2.868	2.145
2020 Q3–2021 Q2	1.686	3.073/2.723	0.541/0.132	3.631	2.621

**Table 2:** Five most central network hubs derived from the degree and eigenvector centrality measures.

	Degree Centrality	Eigenvector Centrality
2015 Q1–2015 Q4	Directors, Policy provisions, Law, Disclosure, Deliberation	Directors, Policy provisions, Law, Disclosure, Deliberation
2016 Q1–2017 Q2	Policy provisions, Law, Disclosure, Directors, Deliberation	Policy provisions, Law, Directors, Disclosure, Deliberation
2017 Q3–2017 Q4	Industry, Society, Guarantee, Reform, Conference	Industry, Society, Guarantee, Healthy, Reform
2018 Q1–2019 Q2	Market, Finance, Risk, Financial bonds, Disclosure	Market, Finance, Disclosure, Financial bonds, Risk
2019 Q3	Market, Finance, Policy provisions, Disclosure, Financial bonds	Market, Policy provisions, Disclosure, Finance, Financial bonds
2019 Q4–2020 Q1	Market, Disclosure, Deliberation, Risk, Contract	Market, Disclosure, Contract, Risk, Deliberation
2020 Q2	Deliberation, Disclosure, Configuration, Market, Contract	Deliberation, Disclosure, Contract, Market, Risk
2020 Q3–2021 Q2	Configuration, Deliberation, Financial bonds, Retirement, Insurance	Configuration, Retirement, Insurance, Financial bonds, Disclosure

**Table 3:** Information on the modules of the linguistic network of 2017 Q3–2017 Q4.

Module ID	% of Nodes	Selected Keywords
1	50.98%	Deliberation; Society; Conference; Economy; Directors; Management construction
2	41.18%	Employment; Science and Technology; Reform; Industry; Innovation
3	7.84%	Distribution; Patient; Doctor; Medicine; Sales

**Table 4:** Topic structure of different phases.

Phase	Topic Structure
2015 Q1–2015 Q4	Topic 4
2016 Q1–2017 Q2	Topic 4
2017 Q3–2017 Q4	Topic 1, 2, 4
2018 Q1–2019 Q2	Topic 3, 4
2019 Q3	Topic 2, 3, 4
2019 Q4–2020 Q1	Topic 2, 3, 4
2020 Q2	Topic 2, 3, 4
2020 Q3–2021 Q2	Topic 3, 4

## Data Availability

The analyzed data is in the public domain and accessible to all researchers. However, we do not have the authority to re-distribute the data.

## Appendix A

**Table A1:** Summary of module detection.

Phase	Modularity	*CC*	Number of Modules
2015 Q1–2015 Q4	0.199	0	1
2016 Q1–2017 Q2	0.3	0	1
2017 Q3–2017 Q4	0.337	0.455	3
2018 Q1–2019 Q2	0.332	0.303	2
2019 Q3	0.503	0.392	5
2019 Q4–2020 Q1	0.504	0.422	4
2020 Q2	0.502	0.559	4
2020 Q3–2021 Q2	0.477	0.541	3

## References

[1] Van Gemert-Pijnen J.E., Wynchank S., Covvey H.D., Ossebaard H.C. (2012). Improving the credibility of electronic health technologies. Bull. World Health Organ..

[2] Yang F., Shu H., Zhang X. (2021). Understanding "Internet Plus Healthcare" in China: Policy Text Analysis. J. Med. Internet. Res..

[3] Ju W., Chen X., Yin W., Yang G., Lin H., Wu J., Fang X. (2021). Evolution and development of E-healthcare policy in China. Digit. Med. China.

[4] He D., Gu Y., Shi Y., Wang M., Lou Z., Jin C. (2020). COVID-19 in China: The role and activities of Internet-based healthcare platforms. Glob. Health Med..

[5] Tu J., Wang C., Wu S. (2017). Using technological innovation to improve health care utilization in China’s hospitals: The emerging ‘online’ health service delivery. J. Asian Public Policy.

[6] Han Y., Lie R.K., Guo R. (2020). The internet hospital as a telehealth model in China: Systematic search and content analysis. J. Med. Internet. Res..

[7] Olper A., Swinnen J. (2013). Mass media and public policy: Global evidence from agricultural policies. World Bank Econ. Rev..

[8] Vargo C., Guo L. (2017). Networks, big data, and intermedia agenda setting: An analysis of traditional, partisan, and emerging online US news. J. Mass Commun. Q..

[9] McCombs M.E., Shaw D.L., Weaver D.H. (2013). Communication and Democracy: Exploring the Intellectual Frontiers in Agenda-Setting Theory.

[10] Guo L., McCombs M. Network agenda setting: A third level of media effects. Proceedings of the Annual Conference of the International Communication Association.

[11] Kamenova K., Reshef A., Caulfield T. (2014). Angelina Jolie’s faulty gene: Newspaper coverage of a celebrity’s preventive bilateral mastectomy in Canada, the United States, and the United Kingdom. Genet. Med..

[12] MacKenzie R., Chapman S., Barratt A., Holding S. (2007). “The news is [not] all good”: Misrepresentations and inaccuracies in Australian news media reports on prostate cancer screening. Med. J. Aust..

[13] Barabas J., Jerit J. (2009). Estimating the causal effects of media coverage on policy-specific knowledge. Am. J. Pol. Sci..

[14] Rachul C., Caulfield T. (2015). The media and access issues: Content analysis of Canadian newspaper coverage of health policy decisions. Orphanet. J. Rare Dis..

[15] Zolnoori M., Huang M., Patten C.A., Balls-Berry J.E., Goudarzvand S., Brockman T.A., Pour E., Yao L. (2019). Mining News Media for Understanding Public Health Concerns. J. Clin. Transl. Sci..

[16] Liu Q., Zheng Z., Chen J., Tsang W., Jin S., Zhang Y., Akinwunmi B., Zhang C.J., Ming W.K. (2021). Health Communication About Hospice Care in Chinese Media: Digital Topic Modeling Study. JMIR Public Health Surveill..

[17] Xu G., Meng Y., Chen Z., Qiu X., Wang C., Yao H. (2019). Research on topic detection and tracking for online news texts. IEEE Access.

[18] Mehler A., Lücking A., Banisch S., Blanchard P., Job B. (2016). Towards a Theoretical Framework for Analyzing Complex Linguistic Networks.

[19] Zhang J., Fan X., Li Y., Ma S. (2022). Heterogeneous graphical model for non-negative and non-Gaussian PM 2.5 data. J. R. Stat. Soc. Ser. C Appl. Stat..

[20] Ma S., Huang J. (2017). A concave pairwise fusion approach to subgroup analysis. J. Am. Stat. Assoc..

[21] Watts D.J., Strogatz S.H. (1998). Collective dynamics of ‘small-world’ networks. Nature.

[22] Barabási A.L., Albert R. (1999). Emergence of scaling in random networks. Science.

[23] Newman M.E. (2010). Networks: An Introduction.

[24] Blondel V.D., Guillaume J.L., Lambiotte R., Lefebvre E. (2008). Fast unfolding of communities in large networks. J. Stat. Mech..

[25] Lambiotte R., Delvenne J.C., Barahona M. (2014). Laplacian dynamics and multiscale modular structure in networks. IEEE Trans. Netw. Sci. Eng..

[26] Mei Q., Zhai C. Discovering evolutionary Topic patterns from text: An exploration of temporal text mining. Proceedings of the Eleventh ACM SIGKDD International Conference on Knowledge Discovery in Data Mining.

[27] Zhang Y., Li T., Chen K., Dai K. (2018). A Review of the Media Impact on Public Policy Process in China: The Failure of “Traditional Media Framework” and the Absence of "Social Media Framework". Glob. Media J..

[28] Tong B. (2008). The Relationship between Mass Media and Public Policy: A Review of “News Media and Micropolitics: The Role of Media in Public Policy”. Contemp. Commun..

[29] Cui B. (2013). "Change" and “Trend” in the Era of Great Media—2013 China Media Development Report. Media.

